# Primary pancreatic tuberculosis mimicking pancreatic body cancer. A case report and review of the literature

**DOI:** 10.1016/j.amsu.2020.08.040

**Published:** 2020-08-29

**Authors:** Seifeddine Ben Hammouda, Amina Chaka, Manel Njima, Ibtissem Korbi, Hanen Zenati, Abdelfatteh Zakhama, Rim Hadhri, Khadija Zouari

**Affiliations:** a Department of Pathology, Fattouma Bourguiba University Hospital, Monastir, Tunisia; b Department of Digestive Surgery, Fattouma Bourguiba University Hospital, Monastir, Tunisia

**Keywords:** Pancreatic tuberculosis, Pancreatic cancer, Pancreatic mass, Anti-Tuberculosis therapy, Histopathology

## Abstract

Isolated pancreatic tuberculosis (PT) is an extremely rare disease, with non-specific clinical characteristics, making the diagnosis often challenging with pancreatic cancers.

Here we report a case of a 36-year-old female, who was admitted to our hospital after suffering from a 3-month history of epigastric abdominal pain, night sweats and weight loss. The physical examination was normal. The radiological findings revealed the presence of a pancreatic mass and multiple abdominal lymphadenopathy, suggestive of malignancy. The initial differential diagnosis suspected was pancreatic tuberculosis. Tuberculosis skin test was performed and was highly positive (>22 mm). Computed tomography (CT)-guided biopsy of peripancreatic lymph node was carried out and the histopathological exam confirmed the diagnosis of PT. Therefore, anti-tuberculous therapy was initiated, leading to clinical and radiological improvement.

The diagnosis of PT is rare and can sometimes be misleading. It should be considered when a pancreatic mass is observed, especially in endemic countries, to ovoid unnecessary interventions.

## Highlights

• Isolated pancreatic tuberculosis is an extremely rare disease, with non-specific clinical characteristics.
• Clinical presentation and radiological findings of a pancreatic mass may be suggestive of malignancy.
• Preoperative microbiological or/and histological diagnosis should be made to avoid the patient unnecessary surgical procedures.

## 1 Introduction

Pancreatic tuberculosis (PT) is an extremely rare condition, even in countries where the disease is highly prevalent. The diagnosis is often challenging as clinical and radiological features can mimic pancreatic cancer [1,2]. The excellent evolution after anti-tuberculous therapy makes it imperative to diagnose pancreatic tuberculosis early to avoid unnecessary surgical procedures. We report one such case of primary PT to emphasize one of more rare causes of pancreatic masses.

## 2 Case report

**Fig. 1:**
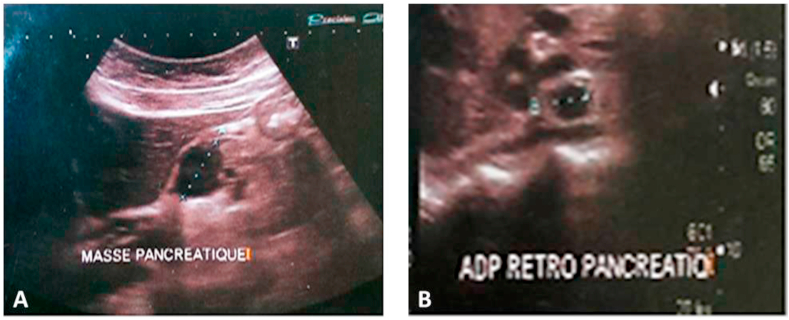
Abdominal ultrasound showing a 40-mm mass in the pancreatic body (A) with multiple peripancreatic lymphadenopathy (B).

**Fig. 2:**
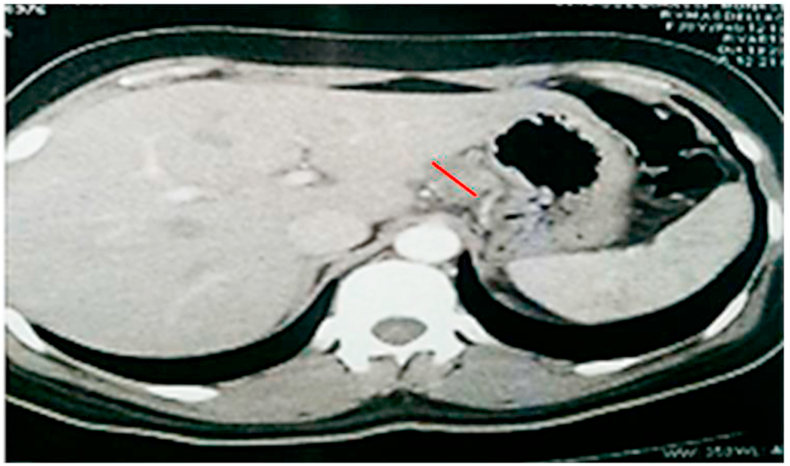
Abdominal Computed tomography (CT) revealed a 45 × 28 mm low-density lesion, arising from the pancreatic body.

We hereby report a case of a 36-year-old female, who was admitted to our hospital after suffering from a 3-month history of epigastric abdominal pain, night sweats and weight loss. She received initially a symptomatic treatment, but with no improvement. Physical examination and biological investigations were normal. An abdominal ultrasound was performed and showed a 40-mm mass in the pancreatic body with multiple peripancreatic and celio-mesenteric lymphadenopathy (Fig. 1). Abdominal Computed tomography (CT) was carried out then and revealed a 45 × 28 mm low-density lesion, arising from the pancreatic body. This lesion had a heterogeneous appearance after the administration of contrast agent. Multiple peripancreatic, mesenteric and hilar lymphadenopathy with a low-density and necrotic appearance were noted (Fig. 2). Chest CT did not show any lesion.

**Fig. 3:**
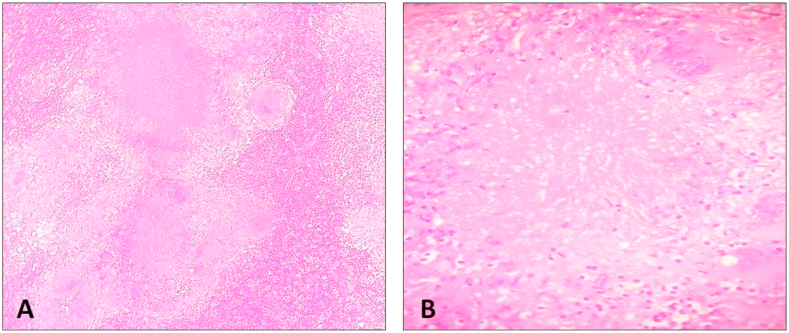
Histological findings, hematoxylin and eosin, showing caseous granulomatous inflammation (A, x100). It corresponds in peripheral rim of epithelioid histiocytes with some multinucleated giant cells surrounding a central granular necrotic region (B, x200).

Based on the above findings the diagnosis of a pancreatic neoplasm with multiple lymph node metastases was suspected. Therefore, the patient was referred to the department of digestive surgery for resection of the pancreatic mass. On examination, the patient was not icteric with no palpable lymphadenopathy. Abdominal examination did not reveal any particular sensitivity or mass. Her laboratory investigations: Complete Blood Count (CBC), renal and liver function were normal. Tumor markers such as CA 19-9 and CEA showed normal levels. Face to this discordance between clinical and radiological findings, other differentiated diagnoses were suspected and included autoimmune pancreatitis and PT. Tuberculosis skin test was highly positive (>22 mm). Computed tomography (CT)-guided biopsy of peripancreatic lymph node was performed. Histopathological findings showed caseous granulomatous inflammation corresponding in peripheral rim of epithelioid histiocytes with some multinucleated giant cells surrounding a central granular necrotic region. There was no evidence of malignancy (Fig. 3). Thus, the diagnosis of isolated PT was given.

The patient was subsequently administrated standard antituberculous treatment, which led to clinical and radiological improvement.

## 3 Discussion

Pancreatic tuberculosis is a rare disease, even in countries where the disease is highly prevalent [1]. It was first studied by Harles in 1912 and most of medical research papers on this rare disease is limited to small case series or case reports [2]. PT usually affects young adults and is seen equally in both male and female patients [2,3]. It is most often associated with immunosuppression or miliary tuberculosis (TB) [4]. Pathogenesis of isolated PT remains poorly understood [5]. PT may produce a variety of clinical presentations and most of reported clinical features of this disease are non-specific [6,7]. Feng Xia et al. have suggested characteristics of PT as follows: “i) mostly occurs in young people, especially female; ii) have a past history of TB, or come from endemic zone of active tuberculosis; iii) often present with epigastric pain, fever and weight loss; iiii) ultrasound and CT scan show pancreatic mass and peripancreatic nodules, some with focal calcification” [8].

The most common symptoms of PT and their frequency, according to a largest review, are summarized in Table 1 [9]. There is no surprise that the clinical feature in PT mimics pancreatic neoplasms. In fact, symptoms such as abdominal pain, anorexia, weight loss, jaundice and a pancreatic mass are suggestive of malignancy and raise strong suspicion of a pancreatic cancer [2,9]. Thus, patients presented with such complaints should be meticulously investigated in order to avoid unnecessary pancreatic resection and the attributed risks.

**Table 1:** Frequency of pancreatic tuberculosis symptoms.

Pancreatic Tuberculosis Symptoms	Frequency
Abdominal pain	66%
Fever/night sweats	52%
Anorexia/significant weight loss	46%
Malaise/weakness	28%
Back pain	20%
Jaundice	15%

Imaging of the pancreas by ultrasound or CT, which are often used for initial investigations, has demonstrated that PT can mimic a pancreatic cancers [10,11].

Ultrasonography (US) shows usually focal hypoechoic lesions or cystic lesions of the pancreas [12]. For CT scan findings, they include irregular borders or diffuse enlargement of the pancreas, hypodense lesions and enlarged peripancreatic lymph nodes [[13], [14], [15]]. The pancreatic mass is presented as a single tissue process in 62.5% of cases and show usually a heterogeneous appearance. It is located often in the head (56%) and is associated with a peripancreatic lymphadenopathy in 75% of cases [[13], [14], [15]]. Invasive diagnostic techniques such as CT/US- guided percutaneous biopsy and surgical biopsy are more reliable and definitive in contrast to noninvasive techniques. In fact, tissue obtained from biopsy can be evaluated for pathologic and microbiological examination [[16], [17], [18]].

Histologically, the presence of caseous granulomatous inflammation and positive stain for acid-fast bacilli are suggestive of tuberculosis. Typical epithelioid and gigantocellular granuloma is found in 60% of cases and rarely caseous necrosis is seen [18,19]. Microbiological examination is used equally to confirm the diagnosis and is based essentially on cultures for mycobacteria, which take up to 6 weeks to grow [20,21]. Besides, it is interesting to emphasize that polymerase chain reaction (PCR) based assay is a highly specific testing and may give a positive result even though cultures of these tissues and special staining techniques are negative [22].

Once the diagnosis is given, the management of PT rest on antitubercular therapy. This treatment comprises multi-drug anti-tuberculous chemotherapy (streptomycin, rifampin, isoniazid, pyrazinamide and ethambutol) and it is usually recommended for between 6 and 12 months [23]. The guidelines of Directly Observed Therapy Short course (DOTS) recommend only six months of therapy even for severe forms of tuberculosis [23,24]. The response to treatment is usually predictable and complete with clinical and radiological improvement. Therefore, longer duration of treatment is unnecessary because it results in higher costs and can exposes patients to more side effects [23]. PT recurrence is rarely described [20] and surgery is performed in case of serious complications like compressions, fistulas and hemorrhages [[19], [20], [21]].

## 4 Conclusion

Isolated pancreatic tuberculosis is a rare disease that require high index of suspicion for diagnosis. Clinical presentation and radiological findings of a pancreatic mass may be suggestive of malignancy resulting in a vastly different therapeutic approach and prognostic implication. This disease should be considered in patients presenting with pancreatic mass, especially in immunocompromised condition. Thus, vigorous efforts should be made to obtain preoperative microbiological or/and histological diagnosis to avoid the patient unnecessary surgical procedures.

## Provenance and peer review

Not commissioned, externally peer reviewed.

## sources of funding

None.

## Ethical approval

Exemption from ethnical approval.

## Consent

The patient accepted the publication of his case report.

## Author contribution

Seifeddine Ben Hammouda and Amina Chaka: data analysis and writing the paper.

Manel Njima and Rim Hadhri: bibliography, coordination and helped to draft the manuscript.

Ibtissem Korbi and Hanen Zenati: specimen contribution and data collection.

Abdelfatteh Zakhama and Khadija ZOUARI: revision

## Registration of research studies

This is not applicable to our case report.

## Guarantor

Seifeddine Ben Hammouda.

## Declaration of competing interest

None.
